# Airborne Influenza A Is Detected in the Personal Breathing Zone of Swine Veterinarians

**DOI:** 10.1371/journal.pone.0149083

**Published:** 2016-02-11

**Authors:** Kate M. O’Brien, Matthew W. Nonnenmann

**Affiliations:** Department of Occupational and Environmental Health, College of Public Health, University of Iowa, Iowa City, Iowa, United States of America; The University of Hong Kong, HONG KONG

## Abstract

The 2009 H1N1 pandemic emphasized a need to evaluate zoonotic transmission of influenza A in swine production. Airborne influenza A virus has been detected in swine facilities during an outbreak. However, the personal exposure of veterinarians treating infected swine has not been characterized. Two personal bioaerosol samplers, the NIOSH bioaerosol sampler and the personal high-flow inhalable sampler head (PHISH), were placed in the breathing zone of veterinarians treating swine infected with either H1N1 or H3N2 influenza A. A greater number of viral particles were recovered from the NIOSH bioaerosol sampler (2094 RNA copies/m^3^) compared to the PHISH sampler (545 RNA copies/m^3^). In addition, the majority of viral particles were detected by the NIOSH bioaerosol sampler in the >4 μm size fraction. These results suggest that airborne influenza A virus is present in the breathing zone of veterinarians treating swine, and the aerosol route of zoonotic transmission of influenza virus should be further evaluated among agricultural workers.

## Introduction

The emergence of the swine-origin H1N1pdm09 influenza A outbreak illustrated the need to understand the processes underlying the antigenic shift and zoonotic transmission of influenza A. Influenza A is a highly contagious respiratory virus with 3,000 to 49,000 deaths, annually.[1,2] The expression of the glycoproteins, hemagglutinin (HA) and neuraminidase (NA), on the viral envelope determines the species specific infection via the sialic acid linked oligosaccharide receptors.[3,4] However, some influenza A variants cross the species barrier.[5–7] Porcine respiratory epithelium express both human and avian specific sialyl-oligosaccharide receptors.[8] Upon the co-infection of human and avian influenza, the reassortment of influenza A viral genome can occur in porcine epithelium and lead to formation of novel variants.[9,10] For example, phylogenetic analysis of circulating influenza A virus among American and Italian swine herds in the 1990s showed that the virus was a reassortment of avian, human, and classic swine viral genome.[11,12] Furthermore, the genome sequencing of H1N1pdm09 influenza A showed that the viral genome was derived from classical swine lineage, Eurasian avian-like lineage, and North American triple reassortment lineage.[6,13] Clearly, reassortment of the influenza virus genome occurs in production animal populations. This reassortment places both human and animal populations at risk for the emergence of a highly pathogenic strain of influenza, as is currently ongoing in the poultry industry with the H5N2 outbreak.[14,15] Therefore, determining routes of zoonotic transmission is critical for the prevention of influenza virus transmission.

Swine influenza is endemic throughout the United States and causes a high morbidity rate among swine herds.[16] At the present time, the influenza A subtypes (H1N1, H3N2, and H1N2) are the most common infection circulating in swine.[16] The zoonotic transmission of swine influenza can occur via the direct, indirect, or airborne route. The direct and indirect route has been well documented, but the airborne route of influenza A infection is not well understood.[17] Corzo et al demonstrated that airborne influenza A particles at a concentration of 3.20X10^5^ RNA copies/m^3^ and 8.58X10^3^ RNA copies/m^3^ can be detected inside the swine facilities and at most 2.1 km downwind of an infected swine herd.[18] Furthermore, aerosolized influenza A virus detection was dependent upon viral shedding in the swine herds’ nasal secretion and density of swine infected.[19] However, these studies collected area air samples from one central location, and did not measure aerosols in swine veterinarians’personal breathing zone.[18,19] Therefore, the personal inhalation exposure of influenza A virus among swine veterinarians needs to be characterized.

Swine workers and their families have a significantly higher risk of influenza A infection than their non-exposed neighbors.[20] Also, swine workers have elevated antibody titers against circulating swine influenza virus variants and higher prevalence of seroconversion than the local communities.[20,21] The protection of agricultural workers and swine from zoonotic transmission of influenza A virus is relevant to both public health and the swine production industry.

The aim of this study was to determine the concentration of influenza A virus in the personal breathing zone of personnel working with infected swine herds.

## Methods

### Sample Population

Two swine veterinarians (veterinarian 1:Farms 1–2 and veterinarian 2:Farms 3–5) were recruited for this study. The veterinarians treated infected swine herds on private farms throughout the State of Iowa. Due to confidentiality, the specific geographical location of the private farms in the State of Iowa cannot be disclosed. Swine veterinarians were called to a swine farm when there was a suspected influenza A infection among the herd. The swine veterinarian took either oral or nasopharyngeal fluid samples. Once a swine herd tested positive for influenza A virus, the veterinarian contacted the research team. This study was carried out with approval from the University of Iowa Institutional Review Board in the Human Subjects Office. Written consent was received from all swine veterinarians that participated in the study.

### Personal sampling of aerosolized influenza A

Study participants were equipped with a backpack containing two aerosol samplers, the National Institute of Occupational and Safety Health (NIOSH) bioaerosol sampler BC251 (NIOSH; Atlanta, GA) and the personal high-flow inhalable sampler head (PHISH),[22,23] and two air pumps, AirChek XR5000 (SKC Inc.; Eighty Four, PA) and Omni (BGI; Waltham, MA). The NIOSH bioaerosol sampler and PHISH were calibrated to an air flow rate of 3.5 L/min and 10 L/min, respectively. The NIOSH bioaerosol sampler contained a 15 mL conical tube (Fisher Scientific; Pittsburgh, PA), a 1.5 mL microcentrifuge tube (Fisher Scientific; Pittsburgh, PA), and a 37-mm, 0.3 μm pore size polytetrafluoroethylene (PTFE) filter (SKC Inc.; Eighty Four, PA). The PHISH is a newly designed sampler that uses standard 37 mm filter material to collect aerosols in the breathing zone that are representative of the inhalable size fraction (d_50_ = 100 μm) at a flow rate greater than other inhalable samplers. For this experiment, the PHISH used a 37-mm, 0.3 μm pore size PTFE filter, which has been recommended for virus aerosol sampling. The NIOSH and PHISH were placed on the study participants’ lapels. During sampling, study participants performed their tasks which included collecting swine oral or nasopharyngeal samples, walking up and down each pen, and observing the behavior of the swine. One integer aerosol sample was collected per bioaerosol sampler per farm. Typically, 30–60 minutes were required to accomplish the oral fluid collection and observation tasks.

### Extraction of influenza virus from sampling media

Hank’s Balanced Salt Solution (HBSS) (Gibco; Waltham, MA) was added to the PTFE filters (5 ml), 15 ml tube (5 ml) and 1.5 ml conical tube (1.5 ml) and vortexed for five minutes at a low speed. Samples were aliquoted and stored at -80°C.

### Collection of swine oral or nasopharyngeal samples

Due to the time difference between the initial evaluation of an infected swine herd and sampling time, the swine veterinarian collected either an oral or nasopharyngeal sample to confirm the presence of influenza A virus infection among the swine herd during sampling. For an oral sample, a cotton rope was hung in the pen. The pigs were allowed to chew on the rope for approximately 30 minutes. The rope was placed in a plastic bag and squeezed to discharge the oral fluids. The oral fluids were then poured into a 50 mL conical tube (Fisher Scientific; Pittsburgh, PA) and placed on ice. For nasopharyngeal samples, the veterinarian placed a flock swab (BD; Sparks, MD) into the nasopharynx and rotated the swab twice. The flock swab was placed into 3 mL of universal viral transport media (BD; Sparks, MD) and placed on ice. Both oral and nasopharyngeal samples were aliquoted and stored at -80°C for further analysis. The criteria for swine to test positive for influenza A virus was a reverse transcriptase real time quantitative polymerase chain reaction (qPCR) Ct value ≤ 37 for the oral or nasopharyngeal swab sample.

### Viral RNA isolation and Quantitative Real-time Polymerase Chain Reaction

Viral RNA was extracted from 1 mL of collected oral, nasal, or aerosol samples using the QIAamp Viral RNA Mini kit (Qiagen; Valencia, CA) per manufacturer’s instructions. Viral RNA was reverse transcribed into complimentary DNA using the SuperScript^®^ Platinum One Step qRT-PCR kit (Life Technologies; Waltham, MA) for a final volume of 25 μL. A 1:4 serial dilution standard curve was generated using influenza A plasmid DNA (Attostar LLC; St. Louis, MN) for qRT-PCR. All samples were run in triplicates. Influenza A primer and probe sequences are as follows: Forward: 5’- GCA CGG TCA GCA CTT ATY CTR AG-3’ Reverse: 5’-GTG RGC TGG GTT TTC ATT TGG TC-3’ and probe: 6FAM-CA GAA TAT ACA “T”CC RGT CAC AAT TGG ARA A-BHQ1.[24] Real-time qPCR was performed using TaqMan reagents (Life Technologies; Waltham, MA) on a QuantStudio 7 Flex (Life Technologies; Waltham, MA) system using the protocol: 50°C for 30 minutes, 95°C for 10 minutes, 45 cycles of 95°C for 15 seconds followed by 55°C for 35 seconds.

### Data analysis

RNA copies were calculated from the samples’ Ct values utilizing the linear regression equation generated from the influenza A plasmid DNA standards. RNA copies per ml were multiplied by the wash volume (1.5 ml or 5 ml) and divided by the total volume of air collected during sampling. The data are reported as geometric mean ± geometric standard deviation. The following equations are examples of the calculations used:

$$Log\;(Viral\;RNA\;copies)=\frac{Ct(sample)-(y-intercept)}{slope}$$

$$Viral\;RNA\;copies/m^{3}=\frac{Viral\;RNA\;copies}{Volume\;of\;air\;sampled}$$

## Results

### Inhalation exposure to influenza virus aerosol

At the time of sampling, the swine veterinarian took either nasopharyngeal or oral samples to verify the presence of influenza A infection among the swine herd. Oral and nasal samples were collected from either swine pens or individual swine that presented with influenza-like symptoms. Oral samples indicate a positive pen containing at most 30 pigs; whereas, a nasopharyngeal swab was collected from individual pigs. Farms 1–4 had at least one oral and nasal sample with a Ct value below the required 37 positive threshold limit; thereby, demonstrating that the swine production facility had infected swine. 46 and 3065 viral RNA copies/mL were detected in oral samples collected from the pen. There were 145920 and 463 RNA copies/mL in nasal swabs collect from individual pigs. Farm 5 did not test positive for influenza A. Three out of the four farms were infected with H3N2 subtype of influenza A. Three farms had natural ventilation (side wall curtains closed) (Table 1).

**Table 1:** Summary of the swine farms during sampling. The criteria for a swine to test positive for influenza A virus was a qPCR Ct value ≤ 37 among the swine oral or nasopharyngeal samples. Data is reported as the average RNA copies/mL of either oral or nasal fluid. All samples were collected during the peak months of influenza A infections. *Room contained 20 sows with 250 piglets. Natural ventilation is not applicable for the sow/nursery farm because the farm is enclosed with solid walls.

Farm	Subtype of influenza A	RNA copies/mL	No. Influenza A qPCR Positive/No.Tested	Number of swine	Type of farm	Month	Outside temperature	Natural ventilation
1	H1N1	46 oral	1/3 pen	3000	Nursery	November	-9°C	Curtains closed
2	H3N2	3065 oral	2/3 pen	2500	Finisher	October	13°C	Curtains open
3	H3N2	145920 nasal	6/7 swine	270*	Sow/ Nursery	April	6°C	Not applicable
4	H3N2	463 nasal	2/2 swine	2400	Finisher	April	8°C	Curtains closed
5	H1N2	Not detected nasal	0/1 swine	2400	Nursery	April	16°C	Curtains closed

The 15 mL conical tube of the NIOSH bioaerosol sampler (particle size selection of >4 μm) collected the majority of viral RNA copies/m^3^ (1743 RNA copies/m^3^). The 1.5 mL microcentrifuge tube (1–4 μm) and the PHISH filter collected similar amounts of viral RNA copies/m^3^ (232 and 545 RNA copies/m^3^). The NIOSH filter (<1 μm) of the NIOSH bioaerosol sampler collected the lowest number of viral particles per volume of air, and influenza RNA viral particles were undetectable after one-week post-diagnosis (Table 2). Farm 5 nasal swab did not test positive for influenza A virus, but 582 and 14 viral RNA copies/m^3^ were collected in the NIOSH 15 mL and 1.5 mL tubes (Table 2). However, only one pig was tested at the time of sampling (Table 1). Also, viable virus was detected in both the NIOSH bioaerosol sampler and the PHISH sampler from Farm 3.

**Table 2:** Influenza A RNA copies/m^3^ of air concentrations detected in the swine facilities using two personal bioaerosol samplers. Personal samples were collected among veterinarians working in swine production facilities that were infected with influenza A (H1N1 or H3N2) virus. Swine veterinarians were called to the swine facilities during a suspected influenza A infection and collected bodily fluids for a diagnosis. Samples were collected at various time during or after this initial evaluation of the infected swine herd. The NIOSH bioaerosol sampler and the PHISH collected samples at a flow rate of 3.5 L/min and 10 L/min, respectively. Total RNA copies/m^3^ were determined by qPCR. The summary of the data is reported as geometric mean (GM) and geometric standard deviation (GSD).

Farm	Time elapsed after initial evaluation	NIOSH 15 mL(> 4μm) (RNA copies/m^3^)	NIOSH 1.5 mL (1–4μm) (RNA copies/m^3^)	NIOSH filter (<1μm) (RNA copies/m^3^)	NIOSH total (RNA copies/m^3^)	PHISH filter (RNA copies/m^3^)
1	0 days	5471	767	70	6309	2481
2	2 days	3491	1478	171	5140	-
3	7 days	3708	1193	Not detected	4901	552
4	14 days	390	35	Not detected	425	119
5*	14 days	582	14	Not detected	596	Not detected
GM(GSD)		1742 (3)	232 (9)	110 (2)	2094 (4)	545 (5)

* RT-qPCR Ct values from all Farm 5 samples were outside the linear range of the calibration curve

## Discussion

To determine the personal inhalation exposure to airborne influenza A virus among swine veterinarians, study participants wore two personal bioaerosol samplers. The NIOSH bioaerosol sampler recovered approximately four times more influenza A RNA viral particles than the novel PHISH sampler (Table 2). We have previously compared these two bioaerosol samplers in chamber trials by aerosolizing H1N1 influenza A. Similar to our chamber experiments (data not shown), NIOSH bioaerosol sampler, with the majority of viral aerosol particles collected in the 1.5 mL microcentrifuge tube (size particle 1–4 μm), recovered more virus than the PHISH. Unlike our chamber experiments (data not shown), the 15 mL conical tube of the NIOSH bioaerosol sampler collected 80% of aerosol influenza A particles in the field (Table 2). Recently, it has been demonstrated that airborne influenza A RNA viral particles can be detected in particle size ranges from 0.4 to 10 μm, with the majority of the RNA viral particles and viable influenza A virus greater than 2.1 μm.[25] This study, along with previous research suggest that airborne influenza A viruses are present in particle sizes greater than 4 μm. [25] However, a more in-depth analysis (*i*.*e*., electron microscopy) is needed to determine the exact particle sizes of aerosolized influenza A virus particles in the swine facilities.

Considering influenza A virion size is 80–120 nm, these results would suggest that airborne influenza A is bound to organic dust or other particulate matter in the swine barn.[4] Organic dust promotes the recruitment of innate immune cells via the upregulation of chemoattractant cytokines (*i*.*e*., interleukin-8 and interleukin-6) in the respiratory tract of both humans and swine.[26–28] The innate immune cells, alveolar macrophages, are critical for the defense against influenza A infection in the swine respiratory tract.[29] H1N1 infected swine depleted of alveolar macrophages had a 40% higher mortality rate than controlled infected swine herds.[29] Interestingly, organic dust reduces macrophage phagocytic activity.[30,31] Thereby, suggesting that organic dust exposure increases the susceptibility of the respiratory tract to viral infection. Viable avian influenza has been detected in dust and other particulates downwind of an infected barn.[32,33] In addition, air samples from a live animal market in Minnesota have tested positive for viable influenza A virus in the pens of infected swine.[34] These results would suggest that swine influenza may be bound to organic dust or other particulate matter and could be transmitted via aerosol. These findings have implications for infection control within swine or other animal production buildings. The findings also have implications for virus transmission to other neighboring animal production buildings, farms, animal production workers and the public. For example, a boy that had contact with swine at a live animal market was infected with influenza A virus that had 99%-100% genomic identity to that of the virus detected in a swine herd.[34] However, additional data are needed to further characterize these virus aerosols and to determine if these virus aerosols have the potential to cause infection.

Swine are considered the “mixing vessel” of influenza A virus, leading to the introduction of novel variants into the general populace (*e*.*g*., 1918 H1N1 and 2009 H1N1).[35] This study suggests that swine workers are inhaling aerosolized influenza virus during the treatment of infected swine and may be the first to be exposed to novel influenza variants. Therefore, swine industry biosecurity practices and the usage of personal protective equipment (PPE) among swine workers is imperative to reduce the risk of zoonotic transmission. Personal protective equipment usage, especially gloves, has shown to decrease seroconverison among swine workers.[36] However, PPE usage is not universally standardized across either small or large farms, and workers may not increase the usage of PPE when they suspect that swine herds are ill. [36] These results from this study suggest that a properly fitted respirator (*e*.*g*., N95) should be worn as a standard operation procedure for swine workers entering a facility that houses swine with an ongoing influenza virus infection. Furthermore, PPE is considered the least effective solution to exposure prevention in the industrial hygiene hierarchy of controls.[37] The development of engineering controls (*e*.*g*., filtration and ultraviolet light) is a more effective solution to reduce influenza aerosols in swine production, and it would decrease inhalation exposure of viral aerosols among swine workers and uninfected animals in swine production.[38] However, aerosolization of influenza A is not the only route of transmission. Therefore, it is imperative that good hygiene practices are observed to reduce direct and indirect transmission of influenza A virus in the swine production facility and the neighboring swine facilities.
